# Book Review of "The Molecular Biology of Cancer" by Stella Pelengaris, Michael Khan (Editors)

**DOI:** 10.1186/1476-4598-6-72

**Published:** 2007-11-16

**Authors:** Christian Schmidt

**Affiliations:** 1 Section of Molecular Genetics and Microbiology and Institute for Cellular and Molecular Biology, University of Texas, 1 University Station, A 5000, Austin, TX 78712, USA; 2 Molecular Cancer, BioMed Central Ltd., Middlesex House, 34-42 Cleveland Street, London W1T 4LB, UK

## Abstract

Here, a review of "The Molecular Biology of Cancer" (Stella Pelengaris and Michael Khan [Editors]) is given. The detailed description of the book is provided here: Pelengaris S, Khan M (Eds): *The Molecular Biology of Cancer*; Blackwell Publishing, Oxford (U.K.); 2006. 531 pages, 214 illustrations, ISBN 9-78140-511-814-9, £31.99.

## 

There are a fair number of books, dealing with cancer biology; unfortunately, they arelimited to certain cancertypes and dated. Here, Pelengaris and Khan familiarize the reader with the cancer cell in Chapter one and selected human cancers in Chapter two. A capturing, comprehensive, clearly written and absolutely accurateintroduction into cancer biology, necessary to understand how tumors evolve, develop and progress is offered in Chapters three through fourteen. This book deserves great praise for the readable presentation of this complex field. Nothing is simplified for the sake of the flow of the text. A brief description of the content of Chapters three to fourteen is presented below.

Chapter three focuses on 'nature and nurture in oncogenesis' with emphasis on the role of environmental carcinogens and the influence of environmental as well as individual risk factors on development and progression of cancers along with a brief introduction into the multi-stage-concept of cancers. DNA replication and cell cycle are discussed in Chapter four. Here, concepts like duplication of DNA, addiction of cancer cells to oncogenes and phases/checkpoints of the cell cycle & currently accepted understanding of signaling cascades, utilized to tightly control the progression through the cell are presented and discussed. In turn, the foundation to comprehend one of the hallmarks of cancer – uncontrolled progression through the cell cycle – is provided. Logically, interested readers find an introduction into the regulation of growth in Chapter five. For example, growth factors and cognate receptors, signaling pathways, cellular senescence, apoptosis and survival are elegantly introduced and woven into an appropriately simplified view with plenty of cited reference for further reading. A more detailed discussion of oncogenes and tumor suppressor genes is the given in the next two Chapters, followed by an expanded introduction into the cell death in Chapter eight. Cellular senescence is then discussed in Chapter nine, beginning with the classical observation of a crisis in a cultured cell population and ending with a briefing on telomeres. Leaving the basic cellular machinery and moving into genetic instability, Chapter ten opens the horizon for caretaker genes and how signaling machines recognize damaged DNA and regulate and tune the repair process. Epigenetic levels, post-transcriptional modifications of mRNA, RNA interference and post-translational modifications of proteins are presented in Chapter eleven with a narrowed view on cancer biology. It is noteworthy that the rapidly emerging field of RNA biology, e.g. micro and piwi-interacting RNA and non-coding RNA is fairly under-represented.

One of the properties of cancer cells is the anchorage-independent growth. Concordantly, the topic, including but not limited to, cell-matrix adhesion, cell-cell interactions and malignancy is discussed in Chapter twelve. Beginning with the architecture of tissues in general, the reader is introduced into the cell biology of the basal membrane, adhesion molecules and, then, the field of cell-cell interactions. With this knowledge, the critical steps in the dissemination of metastases are highlighted, leading to an elegant description of the 'seed and soil' hypothesis. Chapter twelve closes with a perspective on cell invasion. Considering the ever-growing field of cancer immunity research, Chapter thirteen provides an insightful introduction along with a selection of questions, remaining to be addressed. A brief background in innate and adaptive response of the human immune system, including a description of major effector cells, cancer immunosurveillance and immunosuppression and tumor antigens enables a reader to comprehend concepts like antigen-specific therapeutic approaches and the usage of cytokines in cancer management and treatment. How tumor cells invade an immune response is highlighted at the end of Chapter thirteen. Related to the preceding discussion, angiogenesis is introduced in considerable depth in Chapter fourteen; for instance, a discussion of general principles of new vessel growth leads to a reflection on pathological angiogenesis and the role of inhibitors in angiogenesis. Further reading directs to nicely written articles, allowing a more detailed exploration of the accumulating evidence. In sum, the philosophical approach to cancer biology and all related topics discussed in the book, along with well-chosen aphorisms, capture the attention of a reader and stimulate creative reading. Especially pleasing is the construction of chapters into introduction, classical experiments, conclusions and future directions. Study questions and topical suggestions for further readinground the chapter into a rich source of information. Balancing the wealth of molecular and cellular information, it is re-iterated that caring for the cancer patient must be part of any successful attempt to manage or, in the best case, cure this devastating disease. In this context, the book covers aspects of diagnosis and treatment of cancer in order to provide a full view of efforts to understand cancers at the most detailed levels, yielding profound strategies to prevent and cure malignancies. Diagnosis of cancer, its treatment options and the concept of patient care are presented in Chapters fifteen through seventeen. Here, the true synthesis of bench and bedside approaches is marvelously achieved by giving a thoughtful background in the role of molecular pathology in cancer diagnosis and staging, imaging techniques, radio- and chemotherapy, palliative care and the quality of a life at its end with numerous examples of recent developments and practical and ethical issues around cancer research.

The only weakness of the bookis the last chapter, dealing with genomic and proteomic approaches in cancer research and diagnostic. While the chapter in itself is superbly written and of outstanding merit, it could have been incorporated into the Chapters three through fourteen, thus, elevating this book to the status of a classical textbook. Students, preparing for exams, seasoned colleagues and laypersons will find it difficult to ignore and resist the book. As the field progresses and new avenues are opened, this textbook will pass the test of time and remain a companion to the community.

## Competing interests

C.S. is Deputy Editor of *Molecular Cancer *and receives no remuneration for his efforts.

## Authors' contributions

C.S. drafted, finalized and approved of the final version of this article.

